# The impact of delayed sample handling and type of anticoagulant on the interpretation of dysplastic signs detected by flow cytometry

**DOI:** 10.11613/BM.2018.020704

**Published:** 2018-04-15

**Authors:** Bettina Kárai, Zsófia Miltényi, Lajos Gergely, Marianna Száraz-Széles, János Kappelmayer, Zsuzsanna Hevessy

**Affiliations:** 1 Department of Laboratory Medicine, Faculty of Medicine, University of Debrecen, Debrecen, Hungary; 2 Department of Internal Medicine, Faculty of Medicine, University of Debrecen, Debrecen, Hungary

**Keywords:** myelodysplastic syndromes, flow cytometry, pre-analytical error

## Abstract

**Introduction:**

A growing body of evidence supports the usefulness of dysplastic signs detected by flow cytometry in the diagnosis of myelodysplastic syndromes (MDS). Our aim was to assess the impact of pre-analytical variables (delayed sample handling, type of anticoagulant, and different clones of antibody) in the interpretation of flow cytometric results.

**Material and methods:**

Bone marrow samples were labelled and analysed immediately after aspiration and on two consecutive days. The effect of anticoagulant type was evaluated in 16 bone marrow samples. Thirty-seven different immunophenotypic variables were recorded after eight-colour staining. Furthermore, 8 normal peripheral blood samples collected in K_3_-EDTA and Na-heparin were examined with different clones of CD11b antibodies and four parameters were recorded with both anticoagulants on two consecutive days.

**Results:**

Fourteen significant differences were detected in the initial immunophenotype of fresh samples collected in K_3_-EDTA and Na-heparin. Regardless of the anticoagulant type, eleven parameters remained stable despite delayed sample handling. Due to delayed sample processing, more alterations were detected in the samples collected in K_3_-EDTA than in the samples collected in Na-heparin. The type of CD11b clone influenced the reduction of fluorescence intensity only in samples collected in K_3_-EDTA, where the alterations were contrary to the changes observed in Na-heparin.

**Conclusions:**

Delayed sample processing causes considerable immunohenotypic alterations, which can lead to false interpretation of the results. If delayed sample evaluation is unavoidable, markers that remain more stable over time should be considered with more weight in the diagnosis of MDS.

## Introduction

Myelodysplastic syndromes (MDS) are heterogeneous clonal hematopoietic stem cell disorders, therefore no single specific marker or method exists for diagnosing all MDS cases. At the same time, MDS are characterized by variable clinical outcome, which makes precise diagnosis and classification of cases by prognostic category vital. The algorithm currently applied for the diagnosis and prognostic classification of MDS considers several parameters from morphologic and cytogenetic examinations, and in addition, it also recommends the application of other methods, such as flow cytometry (FCM) (*1*). The changes of surface and cytoplasmic antigen expression patterns during normal haematopoiesis are well known (*2*, *3*). In the past few decades several studies demonstrated that there are characteristic alterations compared to normal expression patterns which can support the diagnosis of MDS or MDS-related acute myeloid leukaemia (AML). Relying on the most common dysplastic signs, several flow cytometry scoring systems (FCSS) have been established, and they proved to be even more sensitive than morphology in detecting dysplasia (*4*-*9*). As the feasibility of using FCM for the diagnosis and prognostic classification of MDS got verified, the International/European LeukemiaNet (ELN) Working Group for Flow Cytometry in MDS (IMDSFlow) set the aim of integrating FCM into the recommendations of the World Health Organization (WHO) classification to an even greater extent (*10*). To reach this goal, it is essential to standardize and harmonize the diagnostic and prognostic use of FCM; therefore the recommendations issued by ELN should be followed strictly.

According to the recent ELN recommendation, the immunophenotypic alterations in MDS should be analysed within 24 hours, preferably using bone marrow (BM) samples collected in heparin, or ethylenediaminetetraacetic acid (K_3_-EDTA) as an alternative anticoagulant (*11*, *12*). It is evident that delayed sample handling leads to apoptosis, then necrosis, and it is also known that K_3_-EDTA and sodium(Na)-heparin affect cell viability differently: several studies established that EDTA accelerates apoptosis and necrosis of cells (*13*-*15*). Apoptotic cells exhibit characteristic changes in the nuclear, cytoskeletal, and membrane structure (*16*, *17*). These changes often result in altered expression patterns of markers which resemble dysplastic signs and can cause false interpretation. Apoptosis can be avoided or minimized by adhering to the ELN recommendation, however, the transportation of samples and the resulting delay in sample handling is inescapable in several regional laboratories.

This practical problem motivated us to examine the time-dependent immunophenotypic changes of a range of markers on different cell types and identify the ones which may be mistaken for dysplastic signs. On the other hand – while FCM works fine with samples collected in either K_3_-EDTA or Na-heparin – we also wanted to learn how the application of one or the other type of anticoagulant influenced the expression patterns. Finally, we paid special attention to how different anticoagulant types and clones of antibody used for labelling affected the expression of CD11b, since previous studies published contradictory results in this respect (*18*-*20*).

## Materials and methods

### Study design

Two groups of participants were selected. In the first group twenty-three patients with suspected MDS or myeloproliferative neoplasms (MPN) were included in a prospective study. They were referred to the Department of Laboratory Medicine, University of Debrecen, Hungary, between December 2014 and February 2015 for detailed examination. The clinical and laboratory parameters of patients are summarized in Table 1. Three tubes of bone marrow samples were collected from each patient for diagnostic purposes. One sample was collected in K_3_-EDTA (3.0 mL plastic tubes, ref. 368857; Becton, Dickinson and Company, BD Franklin Lakes, USA) for morphological, flow cytometric and molecular examinations, while two tubes in Na-heparin (6.0 mL plastic tubes, ref. 367876; Becton, Dickinson and Company, BD Franklin Lakes, USA) for cytogenetic analysis. We performed our experiments on residual samples that remained after the diagnostic tests. To examine the effect of the different anticoagulants, we evaluated the *de novo* immunophenotype and its alterations on day 1 and day 2 in samples collected into K_3_-EDTA (N = 23) or Na-heparin (N = 16). Samples were kept on room temperature prior to analysis.

**Table 1:** Clinical and laboratory parameters of patients

	**Patients with suspected MDS or MPN (N = 23)**
Final diagnosis MDS MPN AML Iron, B12 or folate deficiency Other (lymphoma, sepsis)	6 4 3 6 4
Age (years)	60 (23 - 87)
Gender (female/male)	11/12
WBC (x10^9^/L)	5.2 (0.74 - 14.4)
Hb (g/L)	103 (64 - 171)
Plt (x10^9^/L)	206 (11 - 568)
ANC (x10^9^/L)	3.3 (0.59 - 9.16)
WBC - white blood cell count. Hb – haemoglobin. Plt - platelet count. ANC - absolute neutrophil count. MDS - myelodysplastic syndromes. MPN - myeloproliferative neoplasms. AML - acute myeloid leukaemia. The final diagnosis of patients was based on laboratory parameters (*e.g.* B12, folic acid concentrations) as well as morphological, cytogenetic, and flow cytometric examination. WBC, Hb, Plt and ANC parameters were measured in peripheral blood samples of patients with suspected MDS or MPN.	

In the second group residual peripheral blood (PB) samples of eight patients with no haematological malignancy were collected in one tube K_3_-EDTA and one tube Na-heparin for flow cytometry measurements, and they were examined with different clones of CD11b monoclonal antibodies.

We conducted our studies in compliance with the principles of the Declaration of Helsinki. Informed consent was obtained from each participant. The Hungarian Medical Research Council granted permission for our studies (20582-2/2017/EKU).

### Methods

Bone marrow samples were analysed for MDS by eight-colour labelling. The antibodies and clones we examined are shown in Table 2. CD14, CD11b, HLA-DR, CD45, CD64, CD13, CD15, CD34, CD71, CD117, CD300e, CD4, and CD10 markers were purchased from Becton Dickinson Biosciences (San Jose, USA); CD33, CD16, and CD13 markers were purchased from Beckman Coulter, (Brea, USA); CD45 marker was purchased from Invitrogen (Thermo Scientific Inc., Walthman, USA); and HLA-DR marker was purchased from Biolegend (San Diego, USA). Antibody combinations were added to 50 mL BM or PB samples (1 x 10^6^ cells) and incubated for 15 minutes in the dark at room temperature. Then 1 mL lysing solution was added to each tube and samples were incubated for an additional 8 minutes. Finally, samples were washed once in phosphate-buffered saline (PBS) and suspended in 500 mL 1% paraformaldehyde (PFA). The FACS Canto II flow cytometer (Becton Dickinson Biosciences, San Jose, USA) was used for cell analysis. To make the results comparable, the flow cytometer was calibrated daily, using Cytometer Setup and Tracking fluorescent microbeads (Cat No. 641319, Becton Dickinson Biosciences, San Jose, USA) and Autocomp software as recommended by the manufacturer. Data were analysed by FACS Diva version 6.1.3 (Becton Dickinson Biosciences, San Jose, CA, USA) and Kaluza Softwares version 1.2 (Beckman Coulter, Brea, CA, USA).

**Table 2:** Antibody combinations used in flow cytometric examination for the diagnosis of MDS

**Fluorochrome**	**Antibody combination (volume)**		
**MDS diagnosis**		
**FITC**	CD14 (30x) MoP9 (6 μL)	CD15 (20x) MMA (6 μL)	CD71 (16x) LO1.1 (6 μL)
**PE**	CD11b (D12) (6 μL)	123 (SSDCLY107D2) (6 μL)	CD117 (104D2) (6 μL)
**PerCP-Cy5.5/PC5.5**	HLA-DR (L243) (5 μL)	CD34 (8G12) (5 μL)	CD33 (16x) (D3HL60.251) (5 μL)
**PC7**	CD13 (Immu 103.4) (5 μL)	CD13 (Immu 103.4) (5 μL)	CD56 (N901) (5 μL)
**APC**	CD300e (UP H2) (2.5 μL)	CD10 (HI10a) (2.5 μL)	CD34 (581) (2.5 μL)
**APC-AF750**	CD64 (2x) (*22*) (2.5 μL)	CD16 (30x) (3G8) (5 μL)	CD38 (HB7) (2.5 μL)
**PB**	CD4 (16x) (RPA-T4) (5 μL)	HLA-DR (L243) (1 μL)	CD7n (8H8.1) (2.5 μL)
**PO**	CD45 (4x) (HI30) (2.5 μL)	CD45 (4x) (HI30) (2.5 μL)	CD45 (4x) (HI30) (2.5 μL)
	**Intensity of CD11b expression**		
**FITC**	-	CD11b (ICRF44) (6 μL)	-
**PE**	CD11b (D12) (6 μL)	-	-
**PerCP-Cy5.5/PC5.5**	CD33 (16x) (D3HL60.251) (5 μL)	CD33 (16x) (D3HL60.251) (5 μL)	-
**PC7**	CD13 (Immu 103.4) (5 μL)	CD13 (Immu 103.4) (5 μL)	-
**APC**	CD10 (HI10a) (2.5 μL)	CD10 (HI10a) (2.5 μL)	-
**APC-AF750**	CD64 (2x) (*22*) (2.5 μL)	CD64 (2x) (*22*) (2.5 μL)	-
**PB**	HLA-DR (L243) (1 μL)	HLA-DR (L243) (1 μL)	-
**PO**	CD45 (4x) (HI30) (2.5 μL)	CD45 (4x) (HI30) (2.5 μL)	-
MDS – myelodysplastic syndromes. On the basis of the upper panel, not only different cell types can be identified but the characteristic MDS-related signs can also be detected. The intensity of CD11b expression was examined with the help of the lower panel. Monocytes and granulocytes were differentiated on the basis of their side scatter character and CD33, CD64, CD45, and HLA-DR expression.			

In case of MDS examinations, the gating strategy was the following: the first step was elimination of debris with the help of forward scatter (FSC) and side scatter (SSC) bivariate dot plot. Myeloblasts (MBs, CD117+/CD34+/SSCint) and lymphoblasts (LBs, CD117-/CD34+/SSClow) were identified on the basis of SSC character, CD117 and CD34 markers; then CD45 was applied in the last step, back gating. Depending on the SSC character and the intensity of CD45, CD33, CD64, or HLA-DR expression, cells were identified as lymphocytes, monocytes, and granulocytes. CD71+/CD45- cells were classified as erythroid precursors. As for rare events, plasma cells (PCs, CD38 bright) were included in our study (*3*).

Thirty-seven different immunophenotypic variables were recorded for the samples collected in K_3_-EDTA and those ones in Na-heparin for three days (*de novo* = day 0, day 1 and day 2), of which mean fluorescence intensity (MFI) values, robust coefficient of variation (rCV) and percentages of different cell types were calculated daily compared to *de novo* values.

In the second part of our study, we investigated not only the impact of using different anticoagulants on time-dependent changes of CD11b expression on granulocytes and monocytes but also the consequence of using different antibody clones (Table 2). Fluorescein isothiocyanate (FITC) labelled CD11b (clone: ICRF44) was purchased from Sigma Aldrich (Saint Louis, USA), while phycoerythrin (PE) labelled CD11b (D12) was purchased from Becton Dickinson Biosciences (San Jose, USA). The gating strategy was the following: the first step was elimination of debris with the help of FSC and SSC bivariate dot plot. Granulocytes and monocytes were differentiated on the basis of their SSC character and CD33, CD64, CD45, and HLA-DR intensity. Four parameters were recorded: CD11b MFI of the two different antibody clones labelled by different fluorochromes on monocytes and granulocytes in K_3_-EDTA and in Na-heparin right after blood drawing and on two consecutive days.

### Statistical analysis

Considering the low number of samples non-parametric tests were used. Two related groups were compared by Wilcoxon signed-rank test. P < 0.05 was considered statistically significant. In case of time-dependent immunophenotypic changes, where there were more than two related groups, data were analysed by Friedman test. Dunn’s multiple comparison test was applied as *post hoc* test. Statistical analysis and the creation of figures were carried out using SPSS 20.0 (SPSS 20.0, Chicago, USA) and GraphPad Prism 6.0 (GraphPad Software, San Diego, USA) statistical programs.

## Results

### Comparison of FCM parameters of samples collected in K_3_-EDTA and Na-heparin at day 0

When we compared the initial immunophenotype of fresh samples collected in K_3_-EDTA and Na-heparin, we detected significant differences in fourteen parameters indicated in Table 3.

**Table 3:** Immunophenotype changes caused by K_3_-EDTA compared to the Na-heparin sample on day 0 in bone marrow samples

**Cell population**	**Marker**	**Mean fluorescence intensity rates**		**P**
**Na-heparin**	**K_3_-EDTA**
**Granulocyte** **(N = 16)**	SSC	160,346 (133,356 - 177,111)	146,635 (129,971 - 153,327)	0.003
	CD45	3964 (3271 – 4569)	3113 (2696 – 4324)	0.013
	CD15	5743 (4329 – 6828)	6803 (4177 – 7924)	0.134
	HLA-DR	179 (93 – 390)	243 (197 – 286)	0.155
	CD11b	10,389 (7208 - 14,936)	1990 (1593 – 3299)	< 0.001
	CD13	1754 (1398 – 2576)	1786 (1102 – 2288)	0.079
	CD16	1764 (1342 – 2119)	1755 (1395 – 2878)	0.836
	CD10	1215 (977 – 1641)	931 (801 – 1692)	0.179
	CD33	1334 (992 – 1670)	1194 (878 – 1705)	0.044
	CD16%	45.5 (40.1 - 61.3)	47.5 (38.0 - 58.9)	0.641
	CD10%	34.5 (29.2 - 49.5)	34.9 (31.5 - 54.3)	0.733
**Monocyte** **(N = 16)**	SSC	75,758 (68,211 - 80,506)	73,891 (67,578 - 80,301)	0.125
	CD64	1330 (1140 – 2051)	1584 (1105 – 2368)	0.776
	CD4	455 (369 – 543)	496 (323 – 753)	0.036
	CD15	1072 (799 – 1502)	1114 (987 – 1528)	0.433
	HLA-DR	7178 (4578 – 7955)	6165 (4420 – 7139)	0.078
	CD11b	13,545 (11,385 - 20,306)	4001 (3453 – 5080)	0.001
	CD13	3522 (1339 – 7092)	3322 (1106 – 4792)	0.031
	CD14	889 (812 – 1482)	971 (720 – 1254)	0.078
	CD300e	1052 (724 – 1914)	1070 (789 – 1804)	0.955
	CD33	7086 (5827 – 9495)	6790 (5119 – 8390)	0.078
	CD14%	55.4 (41.3 - 62.5)	57.3 (47.8 - 62.8)	0.842
	CD300e%	39.1 (24.2 - 58.8)	41.5 (26.0 - 59.0)	0.272
**Myeloblast** **(N = 16)**	%	0.88 (0.62 - 2.30)	1.06 (0.73 - 2.08)	0.451
	CD45	3571 (3019 – 3951)	3581 (2820 – 3938)	0.877
	CD34	3017 (2276 – 4231)	2495 (1959 – 3125)	0.008
	CD117	2599 (2178 – 5455)	3321 (2805 – 5956)	0.001
**Lymphoblast (N = 7)**	%	0.10 (0.06 - 0.20)	0.08 (0.06 - 0.16)	0.061
**PreB cell (N = 9)**	CD10%	0.50 (0.25 - 0.95)	0.90 (0.40 - 1.11)	0.172
**Lymphocyte** **(N = 16)**	%	9.70 (6.25 - 15.45)	10.30 (6.03 - 14.88)	0.82
	SSC	20,777 (19,017 - 21,783)	20,427 (18,451 - 21,907)	0.352
	CD45	15,833 (12,913 - 18,175)	16,742 (13,839 - 21,026)	0.004
**Erythroid precursor** **(N = 16)**	%	18.80 (10.33 - 31.25)	19.40 (11.83 - 31.25)	0.776
	CD71	6158 (3767 – 8283)	3564 (2201 – 5261)	0.003
	CD71rCV	79.5 (53.8 - 112.3)	102.7 (81.0 - 117.7)	0.049
**Plasma cell** **(N = 15)**	%	0.2 (0.2 - 0.5)	0.5 (0.3 - 0.9)	0.005
	CD38	29,173 (25,738 - 32,208)	35,393 (29,221 - 38,793)	0.002
SSC - side scatter. Mean fluorescence intensity values and percentages (%) are presented as median and interquartile range. The number of cases by cell population is different because certain cell types were not detectable in all samples (sensitivity: 0.03). The groups (K_3_-EDTA and Na-heparin) were compared by Wilcoxon signed-rank test. P < 0.05 values were considered statistically significant.				

Six parameters were significantly higher in samples collected in K_3_-EDTA as compared to samples collected in Na-heparin: MFI of CD4 on monocytes, MFI of CD117 on MBs, MFI of CD45 on lymphocytes, rCV of CD71 on erythroid precursors and percentage and MFI of CD38 on PCs.

Eight parameters were significantly lower: SSC and intensity of CD45, CD11b and CD33 expression on granulocytes, MFI of CD11b, CD13 on monocytes, MFI of CD34 on MBs and MFI of CD71 on erythroblasts. Despite significant alterations in the CD34, CD117 and CD71 expression, the percentage of MBs, LBs or erythroid precursors did not show significant differences between the samples collected in K_3_-EDTA and Na-heparin, so these alterations did not influence the appropriate gating of the different cell types.

### Immunophenotypic alterations of bone marrow samples caused by delayed sample handling

Due to delayed sample processing ten parameters were significantly altered by day 1 in samples collected in K_3_-EDTA, while in samples collected in Na-heparin only four parameters changed (Table 4). Two of these (the MFI of CD117 on MBs and the MFI of CD38 on PCs) proved to be the most sensitive for delayed sample processing. Regardless of the type of anticoagulant, the intensity of these markers fell continuously during the time of observation.

**Table 4:** Time-dependent immunophenotype changes in bone marrow samples collected with K_3_-EDTA and Na-heparin

**Tube type**	**Cell population**	**Marker**	**Mean fluorescence intensity rates**			**P1**	**P2**
**day 0**	**day 1**	**day 2**
**K_3_-EDTA**	**Granulocyte** **(N = 23)**	SSC	153,327 (138,520 – 176,341)	149,831 (134,405 – 172,463)	124,668 (110,787 - 148,576)	> 0.999	< 0.001
		CD45	3000 (2739 – 3753)	4141 (3313 – 4472)	3610 (2787 – 3972)	0.037	> 0.999
		CD15	3900 (2199 – 7126)	4681 (2203 – 6913)	3734 (2473 – 6290)	> 0.999	0.554
		HLA-DR	235 (185 – 285)	269 (233 – 341)	324 (261 – 452)	0.037	< 0.001
		CD11b	2795 (1709 – 3613)	1566 (887 – 2406)	642 (436 – 1082)	0.117	< 0.001
		CD13	1845 (1185 – 2308)	2538 (1875 – 3073)	2607 (2162 – 3296)	0.006	0.001
		CD16	2179 (1491 – 2930)	2102 (1750 – 3000)	1780 (1003 - 2727)	0.554	0.554
		CD10	1183 (847 – 1627)	1178 (1001 – 1768)	976 (807 - 1285)	0.231	0.231
		CD33	1212 (815 – 1717)	1349 (917 – 1773)	1188 (754 – 1678)	0.055	> 0.999
		CD16%	49.0 (42.0 - 60.8)	51.0 (43.0 - 56.2)	50.0 (40.7 - 59.0)	0.029	0.715
		CD10%	38.1 (31.0 - 47.0)	41.0 (34.4 - 49.5)	38.4 (31.7 - 45.0)	0.081	> 0.999
	**Monocyte** **(N = 23)**	SSC	79,099 (71,934 - 83,805)	68,674 (64,864 - 75,911)	62,401 (60,160 - 67,200)	0.117	< 0.001
		CD64	1596 (1107 – 1886)	1441 (1052 – 1757)	973 (732 – 1544)	0.117	< 0.001
		CD4	579 (422 – 691)	512 (341 – 712)	418 (324 – 552)	> 0.999	0.024
		CD15	967 (280 – 1290)	632 (281 – 836)	564 (309 – 924)	0.081	0.024
		HLA-DR	6320 (5487 – 7790)	5779 (4291 – 10,731)	5109 (3969 – 7598)	> 0.999	0.315
		CD11b	4700 (3712 – 6538)	716 (342 – 1026)	325 (209 – 406)	< 0.001	< 0.001
		CD13	4190 (1403 – 6409)	2873 (1156 – 5090)	2928 (2035 – 4448)	0.006	0.024
		CD14	1121 (777 – 1571)	777 (627 – 1023)	556 (377 – 718)	0.037	< 0.001
		CD300e	1423 (1055 – 1787)	767 (556 – 1263)	523 (379 – 925)	0.081	< 0.001
		CD33	6747 (5015 – 8633)	5270 (3750 – 8869)	4045 (3101 – 5355)	> 0.999	< 0.001
		CD14%	60.0 (50.0 - 65.8)	53.0 (40.0 - 59.0)	25.0 (17.0 - 44.0)	0.231	< 0.001
		CD300e%	43.4 (26.5 - 56.0)	31.3 (25.0 - 47.0)	27.7 (13.1 - 36.0)	0.037	< 0.001
	**Myeloblast** **(N = 23)**	%	0.92 (0.73 - 1.47)	0.81 (0.54 - 1.35)	0.76 (0.57 - 0.97)	0.196	0.008
		CD45	3555 (2383 – 3838)	3242 (2286 – 3992)	3034 (2636 – 4113)	0.421	0.315
		CD34	3023 (2262 – 3709)	2976 (1987 – 3484)	2318 (1829 – 3860)	0.554	0.231
		CD117	3148 (2781 – 3745)	2411 (2128 – 3523)	1876 (1532 – 2592)	0.006	< 0.001
	**Lymphoblast** **(N = 12)**	%	0.17 (0.06 - 0.23)	0.15 (0.05 - 0.17)	0.09 (0.06 - 0.16)	0.554	0.459
	**PreB cell** **(N = 17)**	CD10%	0.90 (0.23 - 1.64)	0.50 (0.19 - 1.02)	0.40 (0.16 - 0.70)	0.215	< 0.001
	**Lymphocyte** **(N = 23)**	%	10.9 (6.6 - 16.2)	10.0 (5.7 - 14.7)	8.3 (4.9 - 12.8)	0.554	< 0.001
		SSC	21,410 (19,704 - 22,477)	19,611 (17,655 - 20,994)	18,366 (16,682 - 19,817)	0.055	< 0.001
		CD45	15,962 (14,646 - 19,342)	14,898 (12,197 - 17,206)	13,087 (9894 - 16,167)	0.081	< 0.001
	**Erythroid precursor** **(N = 23)**	%	14.3 (9.2 - 27.7)	14.1 (10.0 - 29.0)	13.9 (9.4 - 28.0)	0.715	> 0.999
		CD71	4896 (2448 – 6486)	3164 (1888 – 4449)	2486 (1792 – 3552)	0.315	0.015
		CD71rCV	95 (80 - 108.9)	88.9 (68.7 - 113.2)	84.4 (63 - 103.4)	> 0.999	> 0.999
	**Plasma cell** **(N = 22)**	%	0.50 (0.32 - 0.70)	0.50 (0.30 - 0.63)	0.46 (0.30 - 0.74)	0.457	> 0.999
		CD38	36,019 (31,070 - 39,660)	28,946 (24,769 - 33,504)	25,467 (21,210 - 31,042)	0.001	< 0.001
**Na - heparin**	**Granulocyte** **(N = 16)**	SSC	160,346 (133,356 – 177,111)	146,104 (125,819 – 176,537)	129,252 (109,198 - 163,244)	> 0.999	0.004
		CD45	3964 (3271 – 4569)	4680 (3576 – 5277)	4039 (3437 – 4859)	0.335	> 0.999
		CD15	5743 (4329 – 6828)	5245 (3720 – 6371)	4398 (2832 – 5335)	0.867	0.024
		HLA-DR	179 (93 – 390)	197 (118 - 302)	290 (171 – 492)	> 0.999	0.024
		CD11b	10,389 (7208 – 14,936)	11,877 (9311 – 16,119)	10,517 (7569 - 13,451)	0.867	> 0.999
		CD13	1754 (1398 - 2576)	2218 (1641 - 2680)	2111 (1869 - 3250)	0.648	0.008
		CD16	1764 (1342 – 2119)	1939 (1229 – 2618)	1467 (643 – 2142)	> 0.999	0.102
		CD10	1215 (977 – 1641)	1222 (924 – 1805)	997 (667 - 1341)	0.867	0.335
		CD33	1334 (992 – 1670)	1531 (1071 – 1891)	1403 (932 – 1827)	0.472	> 0.999
		CD16%	46 (40 – 61)	47 (38 – 59)	40 (31 – 52)	> 0.999	0.014
		CD10%	35 (29 – 50)	37 (30 – 49)	36 (25 – 46)	> 0.999	0.752
	**Monocyte** **(N = 16)**	SSC	75,758 (68,211 - 80,506)	76,178 (70,576 - 81,870)	72,624 (66,219 - 81,805)	> 0.999	> 0.999
		CD64	1330 (1140 – 2051)	1434 (898 - 1966)	1369 (808 – 1803)	0.156	< 0.001
		CD4	455 (369 – 543)	344 (276 - 590)	261 (208 – 421)	0.335	< 0.001
		CD15	1072 (799 - 1502)	751 (593 - 1249)	681 (496 – 829)	0.024	< 0.001
		HLA-DR	7178 (4578 - 7955)	8003 (4743 – 11,516)	7402 (5690 – 8140)	0.472	> 0.999
		CD11b	13,545 (11,385 - 20,306)	10,174 (7165 – 12,797)	7428 (4317 – 12,032)	0.232	< 0.001
		CD13	3522 (1339 – 7092)	2859 (1195 – 5494)	2559 (1530 - 4105)	0.335	0.867
		CD14	889 (812 – 1482)	878 (650 – 1008)	637 (545 – 907)	> 0.999	0.014
		CD300e	1052 (724 – 1914)	1116 (708 – 1528)	783 (644 – 1273)	0.867	> 0.999
		CD33	7086 (5827 – 9495)	5658 (4675 – 8438)	4443 (3817 – 5897)	0.232	< 0.001
		CD14%	55.4 (41.3 - 62.5)	50.5 (38.8 - 59.8)	36.5 (22.3 - 45.5)	> 0.999	< 0.001
		CD300e%	39.1 (24.2 - 58.8)	41.9 (24.5- 50.8)	32.0 (24.0 - 52.3)	> 0.999	> 0.999
	**Myeloblast** **(N = 16)**	%	0.88 (0.62 - 2.30)	0.74 (0.50 - 1.95)	0.73 (0.42 - 2.12)	0.279	0.065
		CD45	3571 (3019 – 3951)	3554 (2594 – 4571)	3484 (2780 – 4096)	> 0.999	> 0.999
		CD34	3017 (2276 – 4231)	2624 (1535 – 3053)	2137 (1680 – 2812)	0.335	0.102
		CD117	2599 (2178 – 5455)	1973 (1355 – 3308)	1524 (1186 – 2572)	0.004	< 0.001
	**Lymphoblast** **(N = 8)**	%	0.09 (0.05 - 0.18)	0.07 (0.03 - 0.14)	0.07 (0.02 - 0.10)	> 0.999	0.508
	**PreB cell** **(N = 9)**	CD10%	0.50 (0.25 - 0.95)	0.52 (0.25 - 0.85)	0.30 (0.17 - 0.70)	> 0.999	0.001
	**Lymphocyte** **(N = 16)**	%	9.7 (6.3 - 15.5)	7.7 (4.5 - 13.2)	6.9 (3.3 - 10.0)	0.279	0.001
		SSC	20,777 (19,017 - 21,783)	17,295 (15,546 - 20,467)	17,232 (15,318 - 18,176)	0.024	< 0.001
		CD45	15,833 (12,913 - 18,175)	12,204 (9853 - 17,172)	9948 (8465 -14,071)	0.472	0.002
	**Erythroid precursor** **(N = 16)**	%	18.8 (10.3 - 31.3)	20.1 (10.8 - 31.5)	19.2 (10.4 - 33.8)	> 0.999	0.867
		CD71	6158 (3767 – 8283)	4367 (2268 - 7238)	3479 (2233 – 7007)	0.156	0.002
		CD71rCV	79.5 (53.8 - 112.3)	82.1 (67.5 - 114.3)	80.9 (61.3 - 107.3)	> 0.999	> 0.999
	**Plasma cell** **(N = 15)**	%	0.20 (0.20 - 0.50)	0.20 (0.10 - 0.50)	0.30 (0.10 - 0.50)	0.166	> 0.999
		CD38	29,173 (25,738 - 32,208)	19,312 (17,781 - 27,495)	17,022 (14,953 - 18,877)	0.032	< 0.001
SSC - side scatter. Mean fluorescence intensity values and percentages (%) are presented as median and interquartile range. P1 - comparison of day 0 and day 1 results. P2 - comparison of day 0 and day 2 results. The number of cases by cell population is different because certain cell types were not detectable in all samples (sensitivity: 0.03). The groups were compared by Friedman test. Dunn’s multiple comparison test was applied as *post hoc* test. P < 0.05 values were considered statistically significant.							

On top of the day 1 changes, similar number of additional alterations were detected by day 2 in the cases of samples collected in Na-heparin and K_3_-EDTA. Fifteen parameters in scope decreased significantly in samples collected in K_3_-EDTA, while three parameters increased and twelve parameters decreased in samples collected in Na-heparin. Nine parameters changed by day 2 regardless of the type of anticoagulant: the SSC on granulocytes, the MFI of CD4, CD64, CD33 on monocytes and the ratio of CD14 positive monocytes, the percentage of preB cells and lymphocytes, the MFI of CD45 on lymphocytes and the MFI of CD71 on erythroid precursors.

The number of stable markers throughout the 2-day period was 12 in samples collected in K_3_-EDTA as compared to 18 in samples collected in Na-heparin. The following eleven parameters were the most stable regardless of the type of anticoagulant: MFI of CD16, CD10, CD33 on granulocytes, the percentage of CD10 positive granulocytes, HLA-DR on monocytes, MFI of CD45 and CD34 on MBs, the percentage of LBs, erythroid precursors and PCs and the rCV of CD71 on erythroid precursors.

### Immunophenotypic changes of CD11b caused by delayed sample handling

**Figure 1:**
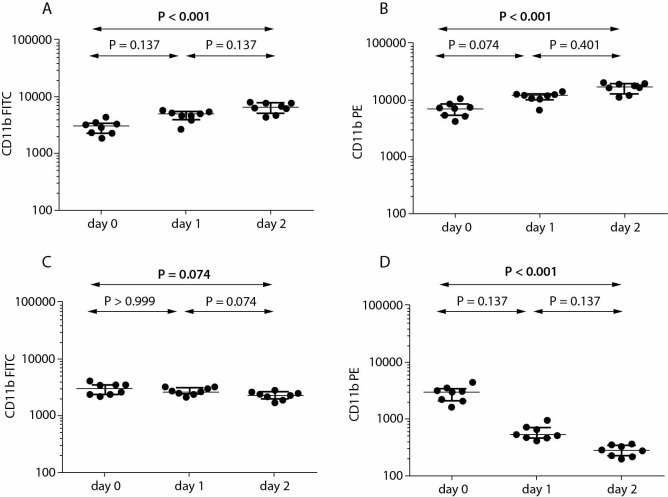
Alterations of CD11b expression on peripheral blood granulocytes caused by delayed sample handling (N = 8). A and B represent samples collected in Na-heparin, C and D represents samples collected in K_3_-EDTA. A and C represent FITC-labelled CD11b, while B and D represent PE-labelled CD11b. The groups were compared by Friedman test. Dunn’s multiple comparison test was applied as *post hoc* test.

**Figure 2:**
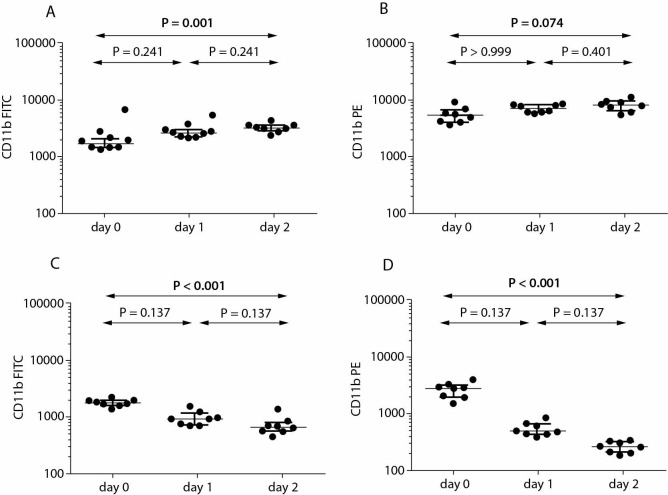
Alterations of CD11b expression on peripheral blood monocytes caused by delayed sample handling (N = 8). A and B represent samples collected in Na-heparin, C and D represent samples collected in K_3_-EDTA. A and C represent FITC-labelled CD11b, while B and D represent PE-labelled CD11b. The groups were compared by Friedman test. Dunn’s multiple comparison test was applied as *post hoc* test.

The intensity of PE-labelled CD11b (clone D12) expression was significantly reduced both on granulocytes (Figure 1D) and monocytes (Figure 2D) in samples collected in K_3_-EDTA by day 2. This decrease was significant in the case of FITC-labelled CD11b (clone ICRF44) only on monocytes (Figure 2C) by day 2. In samples collected in Na-heparin, delayed sample handling caused the opposite phenomenon. Regardless of the type of CD11b clone, the intensity of CD11b expression of granulocytes increased significantly by day 2 in Na-heparin (Figure 1A and 1B). This elevation was significant not only on granulocytes but also on monocytes in the case of FITC-labelled CD11b (clone ICRF44) by day 2 (Figure 2A).

## Discussion

The most important finding of our study was that the type of anticoagulant significantly influenced not only the rate and number of alterations caused by delayed sample handling but also initial expression patterns, where apoptotic cells were absent or were only minimally present. The expression patterns of various cell types proved to be more stable in samples collected in Na-heparin, which supports the recommendation of European LeukemiaNet regarding the preferred type of anticoagulant (*11*, *12*). However, there were some parameters which were altered significantly even by day 1 in samples collected in Na-heparin. In our study, several parameters changed due to delayed sample processing, and unless the examiner is familiar with the extent and direction of such alterations, they could be misinterpreted as dysplastic signs. This can lead to false diagnosis of MDS, or the assignment of already established MDS cases to overly advanced stages.

To the best of our knowledge, we were the first to examine a wide range of markers on different cell types by eight-colour labelling FC method. Previous studies usually focused on the myeloid population and examined a certain marker or only a handful of markers. There is an agreement that some parameters, consistently changed regardless of the type of anticoagulant. One such parameter was the decrease in SSC of granulocytes due to apoptosis, which can be confirmed by morphological examination (*15*). Reduced CD16, CD43 and increased CD45 expression was detected on apoptotic myeloid cells even in isolated white blood cells from citrated, heparinized or EDTA-anticoagulated whole blood (*18*, *20*, *21*). In contrast to the agreement about the behaviour of these markers in the literature, contradictory findings have been published about the effects of apoptosis caused by delayed sample handling on the intensity of CD11b expression: some studies detected a rise in this antigen expression, while others found loss of CD11b (*21*-*23*). These results typically depend on the design of each study. Because the CD11b protein is stored in cytoplasmic granules, the intensity of this marker can markedly increase during activation of neutrophils (*24*). Therefore the first cause which can influence the results is the activation of neutrophils. This may happen during sample processing (purified polymorphonuclear cell, triggered apoptosis) (*25*). However, the results of the study published by Saxton *et al.* suggest that the root cause of activation is not always obvious (*26*). They examined six peripheral whole blood samples anticoagulated in EDTA with or without cell stabilization solution (Cyto-Chexe). They found that the intensity of CD11b expression increased in the case of samples without cell stabilization solution at room temperature during the examined period (4 hour). In contrast, Hodge *et al.* found that Annexin V staining, which can bind to the cell membrane externalized phosphatidylserine (PS) in apoptotic cells, was detected only after 6 hours (*15*). This result suggests that the increased CD11b expression detected by Saxton *et al.* on non-stabilized and non-triggered cells was not caused by apoptosis but rather spontaneous externalization of intracellular CD11b to the cell membrane. So before or during apoptosis the impact of activation should be considered.

We found that CD11b expression changed in different directions over time, depending on the type of anticoagulant: it was over-expressed in samples collected in Na-heparin but decreased in samples collected in K_3_-EDTA. Our results confirmed a second cause which can influence the change of CD11b expression during sample processing, namely, the type of the anticoagulant. Not only cell viability is affected by the type of anticoagulant but also the binding of the antibody to the antigen. Repo *et al.* found that the reason why the type of anticoagulant was able to influence the time-dependent alterations was that CD11b antibodies with D12 clones require divalent cations to bind (*27*). Our results support this explanation, as the MFI values were considerably lower in the presence of the cation chelator EDTA compared to samples drawn into heparin already on day 0 and decreased abruptly by day 1. Other CD11b clones (ICRF44) showed less of a reduction, and the type of clone did not influence the results in the case of samples collected in Na-heparin.

Furthermore, according to our results, the time-dependent changes of CD11b expression depend on sample type. In bone marrow samples CD11b expression on granulocytes did not change in samples collected in Na-heparin, and it decreased significantly on monocytes. In contrast, it increased significantly on granulocytes and monocytes in the peripheral blood samples anticoagulated with Na-heparin. This difference can be explained by the fact that the two examined cell population (neutrophils from peripheral blood and bone marrow) consist of different types of cells with differing amounts of granules and intracellular CD11b storage. On the one hand, normal peripheral blood contains only mature myeloid cells, while bone marrow also contains immature variants. On the other hand, all our peripheral blood samples were normal, while some of the bone marrow samples were pathological.

The majority of markers we examined were not previously included in the studies focusing on the impact of pre-analytical variables. Among these markers, probably the most important was the MFI of CD117, which was altered with both types of anticoagulant. Besides CD34 and CD45, CD117 is routinely used as a gating marker for determining MB count, which is currently the only FCM-based parameter included in the recommendations of the WHO classification (*28*). Despite the obviously diminished intensity of CD117 in all samples on day 1 and day 2, the percentage of MBs was stable by day 1 and altered significantly only by day 2 in samples collected in K_3_-EDTA.

The recently published sensitive and specific Flow Cytometric Scoring System enables FCM to potentially play a larger role in the diagnostic and prognostic work-flow of MDS (*4*-*9*). Flow cytometry can support not only the diagnosis of advanced cases – where there are excess blasts – but also helps in the identification of low-grade cases. For screening purposes, the Ogata score can be used, which contains only four parameters: percentage of MBs in all nucleated cells (MB%), percentage of CD34+ LB in CD34 positive cells, granulocytes SSC/lymphocytes SSC and lymphocyte/MB CD45 MFI ratio (*8*). All parameters suggested by Ogata *et al.* proved to be stable despite a one-day delay in sample handling regardless of the type of anticoagulant. Only the MB percentage decreased significantly by day 2 in samples collected in K_3_-EDTA. Percentage of CD34+ LB in CD34 positive cells was stable in both anticoagulants. The other two parameters (granulocytes SSC/ lymphocytes SSC and lymphocyte/MB CD45 MFI ratio) were adjusted to lymphocytes, which made up the internal control population. In our study, the SSC of both granulocytes and lymphocytes decreased significantly by day 2 regardless of the type of anticoagulant. The MFI of CD45 on MBs was a stable marker, while the MFI of CD45 on lymphocytes decreased significantly with both types of anticoagulants by day 2, which resulted in significant difference in the ratio as well and could cause false interpretation of results on day 2.

Recently a number of studies focused on non-myeloid cell populations like erythroid precursors (*6*, *7*). Although reduced MFI of CD71 was detected in samples collected in either Na-heparin or K_3_-EDTA at day 2, this did not influence the percentage of the erythroid precursors. Furthermore, regardless of the type of anticoagulant, the CD71 rCV, the percentage of LBs, and the MFI of CD45, CD34 on MBs and MFI of HLA-DR on monocytes did not differ significantly even on day 2, which is important because FCS systems rely on several of them (*6*, *8*, *29*).

It must be acknowledged that there are several limitations to the present study. We wanted to examine a homogenous population, but due to the heterogeneous nature of MDS, the patients enrolled with suspected MDS/MPN often ended up with a different final diagnosis. However, the detection of dysplastic signs play a key role not only in the diagnosis of MDS but also in MPN or AML; false interpretation of dysplastic signs on normal cells due to delayed sample processing can cause misdiagnosis of these cases. Furthermore, AML with myelodysplasia-related changes, which is an independent entity in the WHO classification, is associated with poor prognosis. Therefore the detection of dysplastic signs on normal cells or blast population is also important because it influences the treatment. Finally, we could only examine a limited number of cases; therefore further studies with larger samples will be needed to validate our results.

In conclusion, we examined thirty-seven parameters on myeloid, erythroid and lymphoid populations, including mature and immature cell populations. We have already detected alterations in the initial immunophenotype depending on the type of anticoagulant. Because dysplastic signs are identified as alterations compared to the normal pattern, the type of the anticoagulant should always be considered when comparing the samples to the patterns of normal samples. Thus for ease of reference, we recommend that only a single type of anticoagulant is used in any given laboratory.

The pre-analytical error of delayed sample processing can cause considerable immunophenotypic alterations, which can lead to the post-analytical error of false interpretation of the results. Therefore we recommend well-defined standards for sample handling to avoid delays. If the sample needs to be transported to a regional laboratory and delayed sample processing is inescapable, then heparin should be the preferred anticoagulant for flow cytometry, and more stable markers should be weighted more heavily in the diagnosis.

Finally, with respect to choosing a flow cytometry scoring system, it must be noted that the parameters of the Ogata system proved to be stable only for a day. We suggest verifying beforehand how the parameters of the FCSS selected are influenced by the type of anticoagulant and/or delayed sample handling.
